# Identification of Novel 58-5p and *SREBF1* Interaction and Effects on Apoptosis of Ovine Ovarian Granulosa Cell

**DOI:** 10.3390/ijms26020576

**Published:** 2025-01-11

**Authors:** Ruochen Yang, Yong Wang, Sicong Yue, Yueqin Liu, Yingjie Zhang, Chunhui Duan

**Affiliations:** 1 College of Animal Science and Technology, Hebei Agricultural University, Baoding 071000, China; yangruochen2024@163.com (R.Y.); 18833208035@163.com (Y.W.); yueyueyuexiaojuan@163.com (S.Y.); liuyueqin66@hebau.edu.cn (Y.L.); 2 Institute of Feed Research of Chinese Academy of Agricultural Sciences, Beijing 100089, China; 3 Inner Mongolia Academy of Agricultural & Animal Husbandry Sciences, Hohhot 010000, China

**Keywords:** ovine ovarian GCs, PRL, apoptosis, novel 58-5p, *SREBF1*

## Abstract

High concentrations of prolactin (PRL)-induced ovine ovarian granulosa cell (GCs) apoptosis and *MAPK12* could aggravate the induced effect. However, the molecular mechanisms that *MAPK12*-induced GC apoptosis and repressed steroid hormone secretion remain unclear. In this study, GCs in the P group (GCs with high PRL concentration: 500 ng/mL PRL) and P-10 group (GCs with 500 ng/mL PRL infected by lentiviruses carrying overexpressed sequences of *MAPK12*) were collected for whole-transcriptome analysis. Then, we applied the miRNA mimics combined with a dual-luciferase reporter gene assay to explore the molecular mechanisms through which *MAPK12* affected GC apoptosis and steroid hormones secretion. The whole-transcriptome analysis indicated that *MAPK12* regulated high PRL concentration GC apoptosis and steroid hormone secretion mainly through novel 58. The expression of pro-apoptotic proteins Caspase 3 and Bax was increased, while the expression of anti-apoptotic protein BCL-2 declined by novel 58-5p in high PRL concentration GCs (*p* < 0.05); The secretion of steroid hormones and genes associated with steroid secretion (*CYP11A1*, *3β-HSD* and *CYP19A1*) decreased (*p* < 0.05), while the protein expression of the target gene, *SREBF1* of novel 58, was repressed by novel 58-5p in high PRL concentration GCs (*p* < 0.05). Dual-luciferase reporter gene analysis showed that *SREBF1* was confirmed as a target gene of novel 58-5p and the negative feedback interaction was established between novel 58-5p and *SREBF1*. The ggccggctgggggattgccg sequence may be the target site of *SREBF1*, targeted by novel 58-5p. In addition, steroid hormone secretion was reduced and GC apoptosis was suppressed after the interference of *SREBF1* in ovine ovarian GCs with high PRL concentration. In conclusion, novel 58-5p regulated ovine ovarian GC apoptosis and steroid hormone secretion by targeting *SREBF1*.

## 1. Introduction

Sheep (*Ovis aries*) are distinguished physically from monogastric animals for having rumen, and they have a crucial contribution in meeting the high quality animal protein (meat, milk, and skin) production needs of mankind [1]. However, as an important issue, infertility in ewes has caused significant economic losses to the rapid development of the sheep breeding industry [2]. The primary contributors to this condition are factors related to dysfunctional ovulation and compromised steroidogenesis [3,4]. Meanwhile, the utilization rate of mammalian follicles is notably low due to the removal of the majority of follicles from the ovaries before ovulation through a degenerative process known as atresia [5,6]. With advancements in ovarian research, scientists have progressively discerned that the fundamental physiological process governing follicular atresia involves the apoptosis of GCs [7]. In addition, infertility is also commonly accompanied by apoptosis of ovarian GCs [8]. Hence, investigations into follicular degeneration and infertility have progressively centered on the molecular control of apoptosis in GCs. At present, a substantial array of recognized signaling pathways and their enriched genes participate in regulating apoptosis in GCs. Such as P53, toll-like receptor, PI3K-Akt, JAK-STAT, and MAPK signaling pathways [9,10,11,12]. Our previous study found that the *MAPK12* gene of the MAPK signaling pathway, which regulated apoptosis and differentiation, induced apoptosis in ovarian GCs at high PRL concentrations [13]. Despite the continual discovery of novel regulatory elements, understanding the signaling networks involved in GC apoptosis remains limited.

miRNA is a type of endogenous small non-coding RNA with a length of 18–25 nucleotides and a variety of important regulatory roles in cells [14]. Nowadays, it is well known that miRNAs exert important roles in cell proliferation, apoptosis, follicular development, oocyte development, ovulatory dysfunction, steroid hormone secretion, and tumor vascular formation [15]. Sun et al. demonstrated that miR-378 influenced E_2_ production by repressing aromatase translation in porcine GCs and modulating in vitro oocyte maturation by restraining cumulus cell expansion [16]. Moreover, exosomal miR-122-5p promoted the apoptosis of mouse ovarian GCs by targeting the apoptosis-related protein BCL9 [17]. The miR-17-92, miR-183-96-182, and miR-23-27-24 clusters are involved in the apoptosis of GCs and follicular atresia [18]. Lately, although studies have reported interactions between *MAPK12* and miRNA [19], including the demonstrated activation of *MAPK12* that promotes cancer stemness properties by inhibiting microRNA [20], there is no reported evidence on whether miRNA is also involved in the apoptosis in GCs regulated by *MAPK12*. In the present experiment, we identified a previously uncharacterized *MAPK12*-miRNA regulatory pathway that controls GCs apoptosis and steroid hormone secretion under hyperprolactinemia conditions. Whole-transcriptome profiling and functional analyses were integrated to demonstrate that *MAPK12* exerts its regulatory effects through specific miRNAs, representing a novel mechanism in ovulation disorders. These findings establish the first mechanistic link between *MAPK12* and miRNA-mediated regulation in hyperprolactinemia-induced reproductive dysfunction.

## 2. Results

### 2.1. Expression of MAPK12 After Overexpression of MAPK12

**Figure 1:**
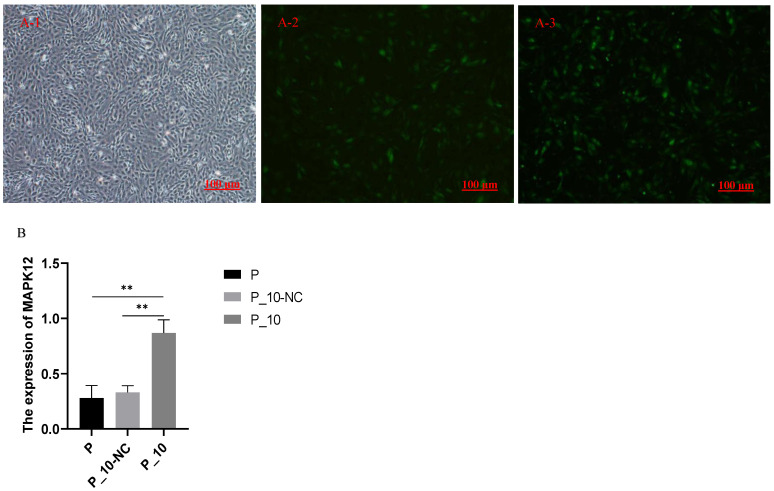
Fluorescence and the expression of *MAPK12* and *P53*. Note: (**A-1**): GCs with 500 ng/mL PRL; (**A-2**): the cell fluorescence of P_10-NC groups; (**A-3**): the cell fluorescence of P_10 groups; (**B**): the expression of *MAPK12* in P, P_10-NC and P_10 groups. “**” indicates *p* < 0.01. P group: GCs with 500 ng/mL PRL; P_10-NC group: GCs in P group were infected by lentiviruses with pGWLV10-new vector (negative control group); P_10 group: GCs in P group were infected by lentiviruses carrying overexpressed sequences of *MAPK12*.

**Figure 2:**
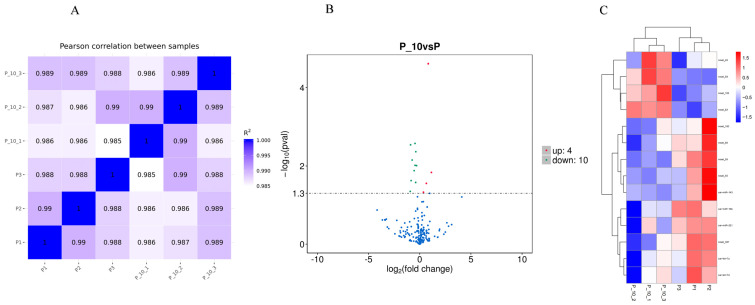
Sample correlation and miRNA differential expression analysis. Note: (**A**): Pearson correlation between samples; R^2^: squared Pearson’s correlation coefficient. The closer the correlation coefficient is to 1, the higher the similarity of expression patterns between the samples. (**B**): Volcano map of differentially expressed genes between the P and P_10 groups; the *x*-axis indicates the expression multiple changes of miRNA between different comparison combinations (log2FoldChange), and the larger the absolute value of the *x*-axis indicates the larger the expression multiple changes between the two comparison combinations. The ordinate indicates the significance level of expression difference. The horizontal dotted line in the figure corresponds to the *p*-value = 0.05 significant difference threshold; The up-regulated miRNAs are represented by red dots, the down-regulated miRNAs are represented by green dots, and the blue dots represent miRNAs with no significant changes. (**C**): Cluster heat map of differentially expressed genes between the P and P_10 groups; the red block indicates high miRNA expression and the blue block indicates low miRNA expression. P group: GCs with 500 ng/mL PRL; P_10 group: GCs in the P group that were infected by lentiviruses carrying overexpressed sequences of *MAPK12*.

**Figure 3:**
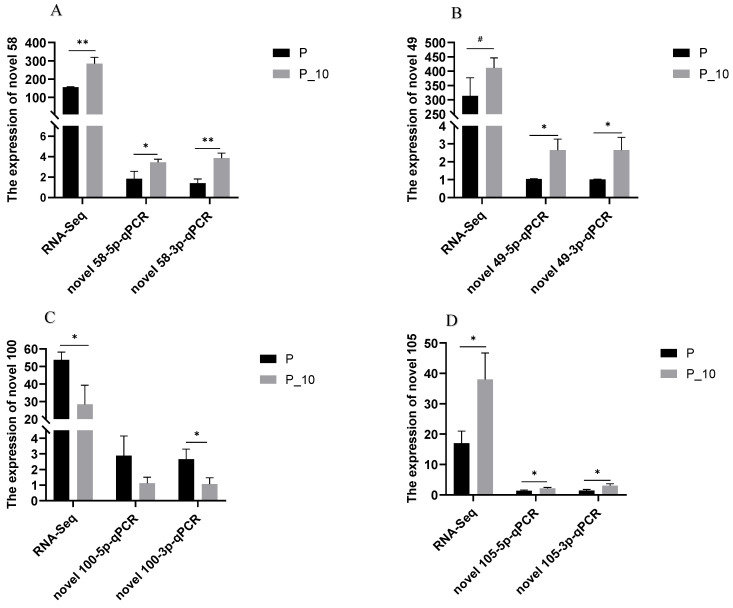
qPCR validation of RNA-Seq. Note: (**A**–**D**): Four novel miRNAs. “^#^”, “*”, and “**” indicate 0.05 < *p* < 0.1, 0.01 < *p* < 0.05, and *p* < 0.01, respectively. P group: GCs with 500 ng/mL PRL; P_10 group: GCs in the P group were infected by lentiviruses carrying overexpressed sequences of *MAPK12*.

The cell fluorescence of P-10-NC (the lentiviruses containing overexpressed *MAPK12* sequences were used to infect GCs, and the lentiviruses with pGWLV10-new vector were used to infect the GCs as the negative control group) and P-10 (GCs treated with 500 ng/mL PRL were infected by lentiviruses carrying overexpressed sequences of *MAPK12*) groups after overexpression is shown in Figure 1(A-2, A-3). The successful transfection was confirmed by observing green fluorescence following the transfection process. RT-qPCR was performed for the transfected P-10-NC and P-10 (Figure 1B) groups. The *MAPK12* expression was remarkably higher (*p* < 0.01) in the P-10 group relative to the P-10-NC and P groups, while there was no significant difference in the expression of *MAPK12* between the P-10-NC and P groups (*p* > 0.05), indicating the success of overexpression.

### 2.2. Small-RNA Identification and the Analysis of DE miRNAs

#### 2.2.1. Small-RNA Identification

Following the elimination of reads containing poly-N, those with 5′ adapter contaminants, lacking 3′ adapters or insert tags, comprising poly A, T, G, or C, and exhibiting low-quality reads from the raw data, a total of 68,794,569 raw reads and 68,054,190 clean reads were generated, and the data after filtering is summarized in Table S1.

#### 2.2.2. The Analysis of DE miRNAs

Correlation analysis using the Pearson method was conducted to assess the consistency of samples. The Pearson’s correlation coefficient (Figure 2A) revealed a substantial similarity among the samples.

The volcano map and heat map of the DE miRNAs are shown in Figure 2B and Figure 2C, respectively. According to the criteria of the *p*-value < 0.05, relative to the P group, 10 miRNAs were significantly downregulated, and 4 miRNAs were significantly upregulated in the P-10 group among all credible miRNAs. The relevant information for the DE miRNAs is shown in Table S2.

#### 2.2.3. RT-qPCR Confirmation

qPCR detection of novel 49-3p, novel 49-5p, novel 58-3p, novel 58-5p, novel 100-3p, novel 100-5p, novel 105-3p, and novel 105-5p in the P and P-10 groups was performed. The results confirmed the DE miRNAs (novel 49, novel 58, novel 100, and novel 105) between the P and P-10 groups, which followed the results acquired from the RNA-Seq (Figure 3). Thus, RNA-Seq can supply accurate results for DE miRNA investigations.

### 2.3. Functional Enrichment Analysis

#### 2.3.1. GO Functional and KEGG Pathway Analysis

**Figure 4:**
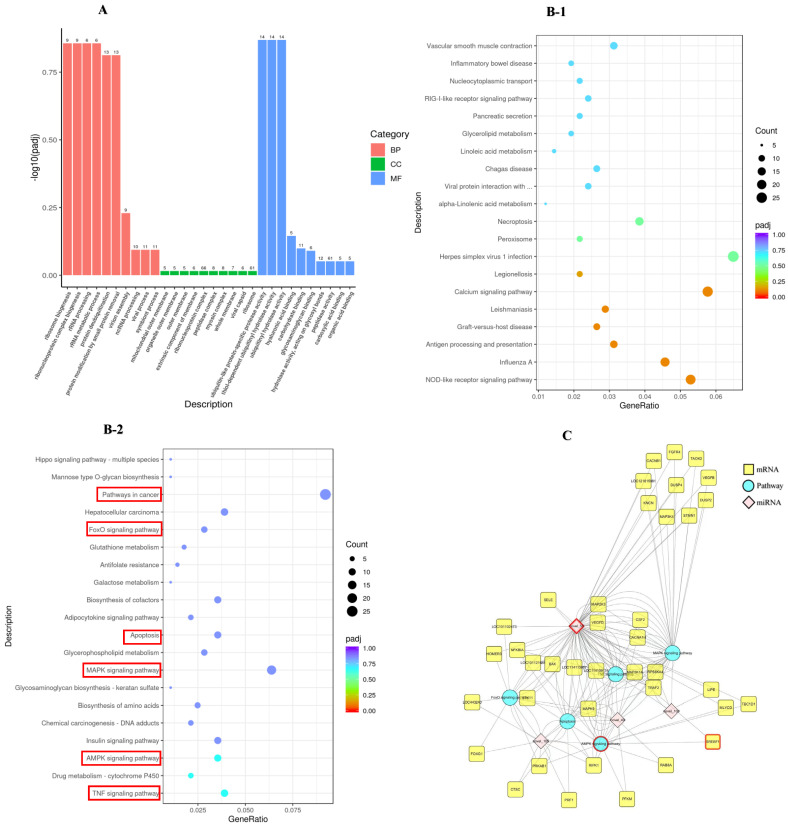
Analysis of the RNA-Seq. Note: (**A**): GO function enrichment of DE miRNAs (the numbers at the top of the bars) between the P and P_10 groups; the *X*-axis is the GO term, and the *y*-axis is the significance level of the GO term enrichment, represented by −log10(padj). Different colors indicate the different functional categories; count: the number of differential target genes annotated on the GO term. (**B-1**,**B-2**): The dot plot of the top 20 downregulated (**B-1**) and upregulated (**B-2**) pathways ranked based on the *p*-value. (**C**): The miRNAs-mRNAs-KEGG pathway interaction network; blue represents pathways, yellow represents mRNAs, and pink represents miRNAs. DE: differential expression; BP: biological processes; CC: cellular component; MF: molecular function. P group: GCs with 500 ng/mL PRL; P_10 group: GCs in the P group were infected by lentiviruses carrying overexpressed sequences of *MAPK12*.

To gain a comprehensive insight into the DE miRNAs, the enrichment of the target gene candidates of DE miRNAs, according to GO and KEGG pathways, was detected. Consequently, 874 GO terms enriched for target gene candidates of DE miRNAs consisted of BP, CC, and MF for which the number was 470, 118, and 286, respectively. Several GO terms were detected to be significantly enriched for the target gene candidates of DE miRNAs, as shown in Figure 4A.

Significantly enriched pathways in target gene candidates of miRNAs were executed according to the KEGG database. The results showed that these target gene candidates of miRNAs were enriched in 317 signaling pathways. The dot plot of the top 20 downregulated (B-1) and upregulated (B-2) pathways are depicted in Figure 4B according to their *p*-value rankings. We found that the target gene candidates of miRNAs were significantly enriched in apoptosis-related pathways, such as the TNF signaling pathway (*p* = 0.005), AMPK signaling pathway (*p* = 0.007), MAPK signaling pathway (*p* = 0.026), apoptosis (*p* = 0.030), and FoxO signaling pathway (*p* = 0.055).

#### 2.3.2. miRNAs-mRNAs-KEGG Pathway Interaction Network

To explore the roles of miRNA in regulating apoptosis, we established an interaction network encompassing miRNAs-mRNAs-KEGG associated with apoptosis (Figure 4C). In the present study, we found that novel 58 can regulate target gene candidates to participate in apoptosis, MAPK signaling pathway, AMPK signaling pathway, TNF signaling pathway, and other pathways closely related to cell apoptosis and steroidogenesis. *SREBF1* (*p* = 0.06) is the target gene with the most significant difference targeted by novel 58. Therefore, novel 58 and the target gene *SREBF1* were selected for the subsequent experiment.

### 2.4. The Effects of Novel 58 on Apoptosis in High PRL Concentration GCs

#### 2.4.1. SREBF1 and Apoptosis-Related Proteins Expression Analysis

**Figure 5:**
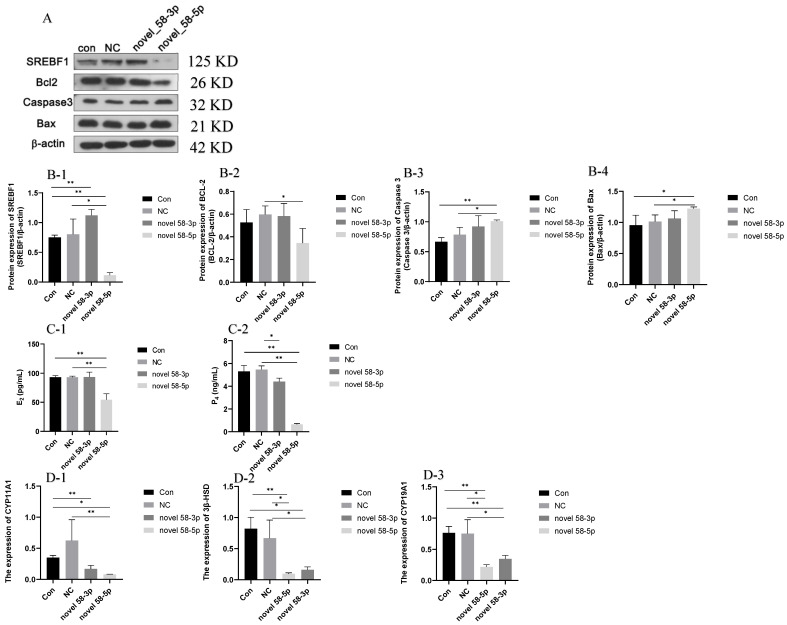
Steroid hormone secretion and related gene expression. Note: (**A**): Western blotting result; (**B-1**–**B-4**): the expression of SREBF1 (**B-1**), BCL-2 (**B-2**), Caspase 3 (**B-3**), and Bax (**B-4**); (**C-1**,**C-2**): the secretion of E_2_ (**C-1**) and P_4_ (**C-2**); (**D-1**–**D-3**): the expression of *CYP11A1* (**D-1**), *3β-HSD* (**D-2**), and *CYP19A1* (**D-3**). “*” and “**” indicate 0.01 < *p* < 0.05 and *p* < 0.01, respectively. Con group: GCs with 500 ng/mL PRL; NC group: GCs in the Con group transfected with the negative control of mimics; novel 58-3p group: GCs in Con group transfected with novel 58-3p mimics; novel 58-5p group: GCs in Con group transfected with novel 58-5p mimics.

The results of SREBF1 and apoptosis-regulated protein expression are presented in Figure 5A,B. The protein expression of SREBF1 decreased in the novel 58-5p group (*p* < 0.05) compared with the con and NC groups, while the protein expression of SREBF1 was increased in the novel 58-3p group (*p* < 0.05) compared with the Con group. This result showed that novel 58-5p could downregulate the expression of SREBF1. As for apoptosis-related proteins, the expression of pro-apoptotic proteins, such as Caspase3 and Bax in the novel 58-5p group increased significantly compared with the con and NC groups (*p* < 0.05), whereas the expression of the anti-apoptotic protein BCL-2 declined in the novel 58-5p group relative to the NC group (*p* < 0.05). The findings suggested that cellular apoptosis might be induced by novel 58-5p in high PRL concentration GCs.

#### 2.4.2. Steroid Hormone Secretion and Related Gene Expression

The secretion of E_2_ and P_4_ was presented in Figure 5(C-1,C-2), exhibited a significant decrease (*p* < 0.01) in the novel 58-5p group compared to the con and negative control (NC) groups. Meanwhile, no significant differences were observed in the secretion of E_2_ and P_4_ between the con and NC groups. The secretion of P_4_ was lower in the novel 58-5p group (*p* < 0.05) than in the NC group. The results of the *CYP11A1*, *3β-HSD*, and *CYP19A1* expression in the con, NC, novel 58-3p, and novel 58-5p groups are shown in Figure 5(D-1–D-3). The expression of *CYP11A1*, *3β-HSD*, and *CYP19A1* was decreased in the novel 58-5p group (*p* < 0.05) related to the con and NC groups. The expression of *3β-HSD* and *CYP19A1* were repressed in the novel 58-3p group (*p* < 0.05) compared with the con and NC groups while the expression of *CYP11A1* (*p* < 0.05) was lower in the novel 58-3p group than in the NC group. These results suggest that the secretion of steroids may be inhibited by novel 58-5p in high PRL concentration GCs.

### 2.5. Analysis of Dual-Luciferase Reporter Gene

**Figure 6:**
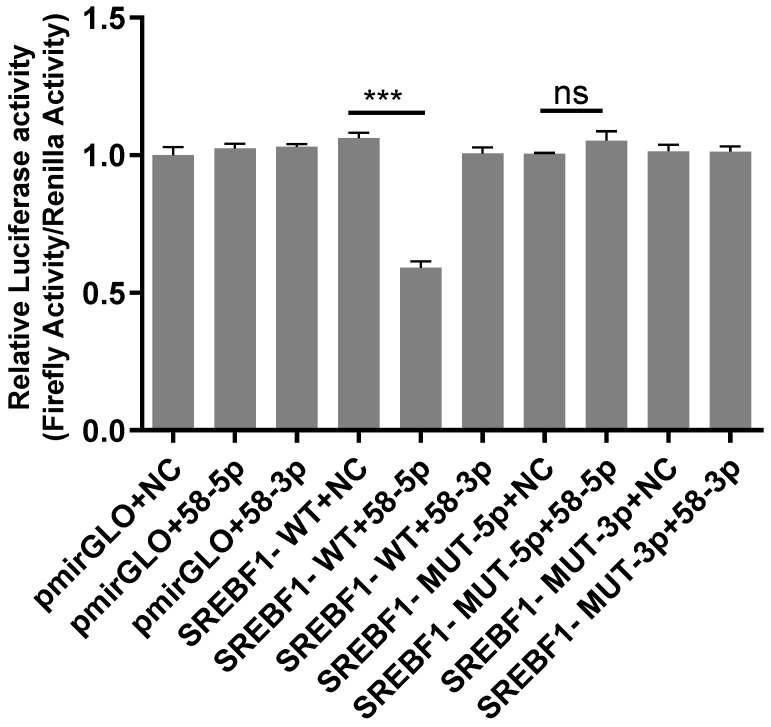
Analysis of the dual-luciferase reporter gene. Note: “***” indicates *p* < 0.001. pmirGLO+NC group: empty plasmid; pmirGLO+58-3p group: pmirGLO was established with novel 58-3p; pmirGLO+58-5p group: pmirGLO was established with novel 58-5p; SREBF1-WT+NC group: wild type SREBF1; SREBF1-WT+58-3P group: wild type SREBF1 was established with novel 58-3p; SREBF1-WT+58-5P group: wild type SREBF1 was established with novel 58-5p; SREBF1-MUT-5P+NC group: mutate SREBF1 at the 5p terminal binding site; SREBF1-MUT-5p+58-5p group: mutant SREBF1 was established with novel 58-5p; SREBF1-MUT-3p+NC group: mutate SREBF1 at the 3p terminal binding site; SREBF1-MUT-3p+58-3p group: mutant SREBF1 was established with novel 58-3p.

The result of the dual-luciferase reporter gene is shown in Figure 6. Compared with the SREBF1-WT+58-5p group, the luciferase activity of the SREBF1-WT+58-5p group significantly declined (*p* < 0.01), demonstrating that novel 58-5p may target regulating SREBF1. The luciferase activity in the SREBF1-Mut-5p+NC group had no significant difference compared to the SREBF1-MUT-5p+58-5p group, indicating that novel 58-5p no longer targeted SREBF1 after the ggccggctgggggattgccg site was mutated. These results suggested that ggccggctgggggattgccg may be the target site of SREBF1 by novel 58-5p.

### 2.6. Bioanalysis of SREBF1

**Figure 7:**
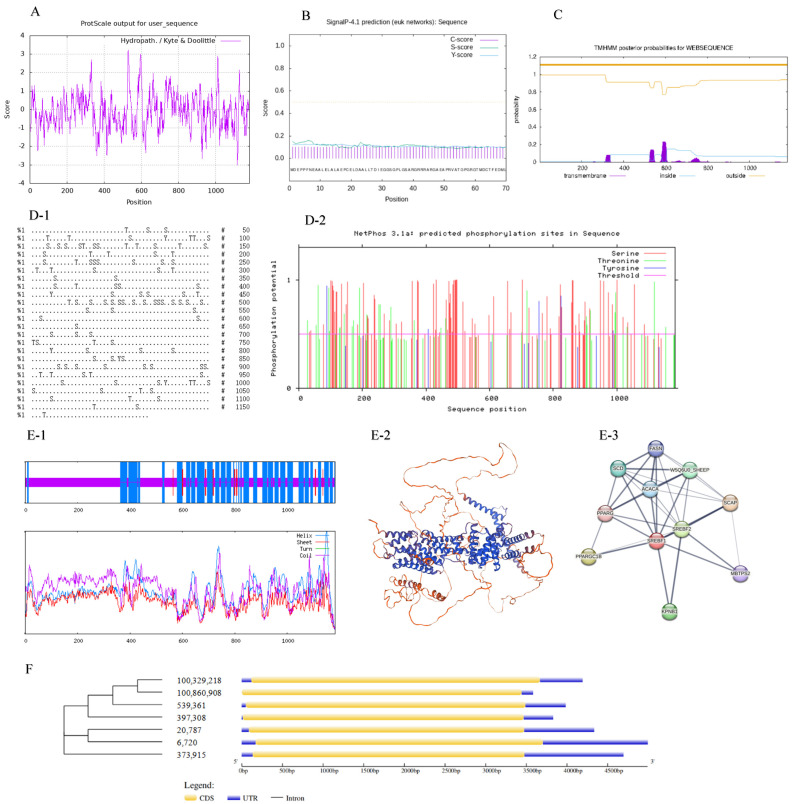
Bioanalysis of *SREBF1*. Note: (**A**): prediction of hydrophobicity of protein; (**B**): prediction of protein signal peptide. The dotted line represents the threshold for prediction; (**C**): prediction of protein transmembrane region; (**D-1**): prediction of phosphorylation site of ovine SREBF1. S represents for Serind, T represents for Threonine, Y represents for Tyrosine; (**D-2**): prediction of phosphorylation site score of ovine SREBF1; (**E-1**): the secondary structure of SREBF1; (**E-2**): tertiary structure of SREBF1; (**E-3**): protein interaction network; (**F**): analysis of ovine *SREBF1* gene structure.

The ProtScale program on the ExPASy server was used to predict the hydrophobicity of the protein encoded by Ovis *SREBF1* (Figure 7A). The highest hydrophobicity score of SREBF1 was at the 531st amino acid (3.222), and the lowest was at the 1118th amino acid (−3.0). The mean value for the hydropathicity of SREBF1 was −0.625, indicating that Ovis SREBF1 belongs to hydrophilic protein. The SignalP 4.1 online software was adopted to predict the signal peptide of Ovis SREBF1. Figure 7B highlighted that the signal peptide scores of Ovis SREBF1 were all less than 0.5, indicating that there was no signal peptide region in Ovis SREBF1. In addition, transmembrane region analysis displayed that there was no transmembrane protein in SREBF1 (Figure 7C). These results indicated that SREBF1 is an intramembrane protein.

The results of serine, threonine, and tyrosine phosphorylation sites (D-1) and phosphorylation site scores (D-2) in SREBF1 are shown in Figure 7D. Phosphorylation occurred when the site score was higher than the threshold value of 0.5, therefore, the serine phosphorylation site is the most, followed by the threonine phosphorylation site, while the tyrosine phosphorylation site is the least. The secondary protein structure of SREBF1 (Figure 7(E-1)) included an alpha helix, extended strand, and random coils at proportions of 38.21%, 1.78%, and 60.02%, respectively, whereas the tertiary structures of the SREBF1 encoding proteins were mainly composed of extended fragments and random coils (Figure 7(E-2)). The forecast results are consistent with the secondary structures. The analysis of the Ovis SREBF1 interaction (Figure 7(E-3)) showed that SREBF1 interacted with SREBF2, ACACA, PPARG, KPNB1, MBTPS2, and other proteins.

The genetic structure of *SREBF1* from multiple species was analyzed using the GSDS2.0 software. The structure of *SREBF1* in 7 different species is similar (Figure 7F), suggesting that *SREBF1* is relatively conserved.

### 2.7. Apoptosis and the Secretion of Steroid Hormones After Interference and Overexpression of SREBF1 in GCs with High PRL Concentration

**Figure 8:**
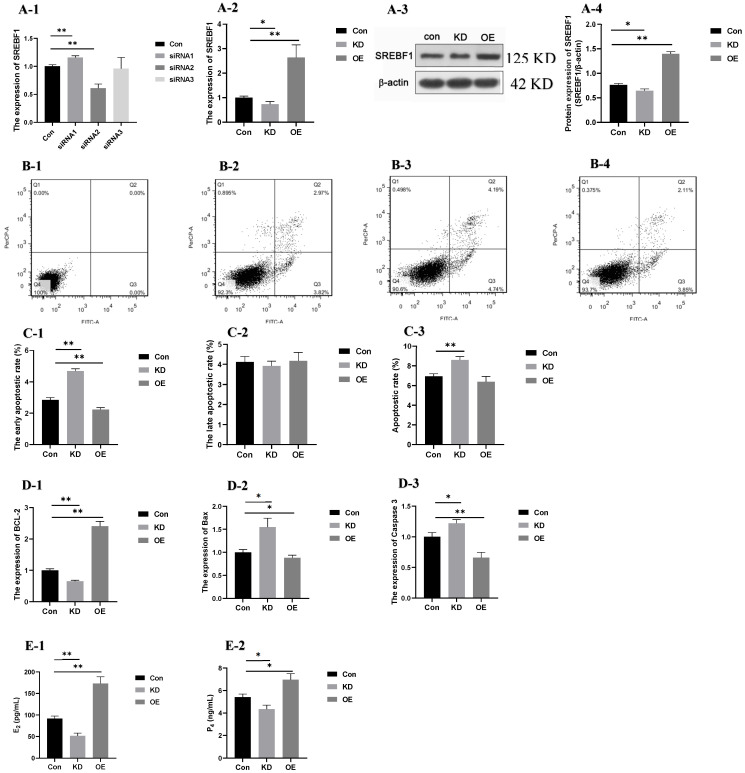
Functional validation of *SREBF1*. Note: “*” and “**” indicate 0.01 < *p* < 0.05 and *p* < 0.01, respectively. (**A-1**): The expression of *SREBF1* after interference of siRNA1, siRNA2, and siRNA3; (**A-2**): the expression of *SREBF1* in the con, KD, and OE groups; (**A-3**,**A-4**): the protein expression of SREBF1 in the con, KD, and OE groups; (**B-1**–**B-4**): apoptosis of the negative control, con, KD, and OE group; (**C-1**–**C-3**): the early apoptositic rate (**C-1**), late apoptositic rate (**C-2**), and total apoptositic rate (**C-3**); (**D-1**–**D-3**): the expression of BCL-2 (**D-1**), Bax (**D-2**) and Caspase 3 (**D-3**); (**E-1**,**E-2**): the secretion of E_2_ (**E-1**) and P_4_ (**E-2**). (**B-1**–**B-4**): The *x*-axis represents PI fluorescence; *y*-axis represents Annexin-V fluorescence. Q1 represents normal dead cells; Q2 represents late apoptotic or necrotic cells; Q3 represents early apoptotic cells; Q4 represents normal cells. Con group: GCs with 500 ng/mL PRL; KD group: GCs in Con group transfected with the interference sequence *SREBF1*; OE group: GCs in Con group transfected with the overexpressed sequence of *SREBF1*.

The results for *SREBF1* expression after interference of siRNA1, siRNA2, and siRNA3 are shown in Figure 8(A-1). The expression of *SREBF1* was significantly lower (*p* < 0.01) after interference by siRNA2. Therefore, GCs with high concentrations of PRL transfected with siRNA2 were selected as the KD group. The gene and protein expressions of *SREBF1* in each group are shown in Figure 8(A-2–A-4). The gene and protein expressions of *SREBF1* were significantly lower (*p* < 0.05) in the KD group than in the Con group, while the gene and protein expressions of *SREBF1* in the OE group was significantly higher than that in the Con group (*p* < 0.01).

#### 2.7.1. Apoptostic Rate and the Expression of Apoptosis-Related Genes

The dead, late apoptotic or necrotic, early apoptotic, and normal cells were distinguished by the detection results (Figure 8(B-1–B-4)). The early apoptotic rate (Figure 8C1) of GCs in the Con group was higher than that in the KD group (*p* < 0.01), and lower than that in the OE group (*p* < 0.05). No differences were observed among the Con, KD and OE groups of the late apoptotic rate (Figure 8C2). The apoptotic rate in the Con group declined significantly compared with the KD groups, while no difference was observed between the Con and OE groups (Figure 8C3).

The expression of apoptosis-related genes (*Bcl-2*, *Bax,* and *Caspase3*) in GCs with high PRL concentration after interference and overexpression of *SREBF1* is shown in Figure 8(D-1–D-3). The expression of the anti-apoptotic gene *Bcl-2* was lower in the KD group than in the Con group (*p* < 0.01) and the expression of the pro-apoptotic gene *Bax* and *Caspase 3* was higher in the KD group than in the Con group (*p* < 0.05). The expression of the anti-apoptotic gene *Bcl-2* in the OE group was significantly higher than that in the Con group (*p* < 0.01), whereas the expression of the pro-apoptotic genes *Bax* and *Caspase 3* decreased in the OE group compared with the Con group (*p* < 0.05). The results indicated that cell apoptosis could be inhibited by overexpression of *SREBF1* in GCs with a high PRL concentration.

#### 2.7.2. The Secretion of Steroid Hormones (E_2_ and P_4_)

The secretion of steroid hormones is shown in Figure 8(E-1,E-2). The secretion of E_2_ and P_4_ was significantly lower (*p* < 0.05) in the KD group and the secretion of E_2_ and P_4_ was increased significantly (*p* < 0.01) in the OE group. These results indicate that the secretion of steroid hormones could be promoted by the overexpression of *SREBF1* in GCs with high PRL concentrations.

## 3. Discussion

In this experiment, we demonstrated that novel 58-5p significantly suppressed steroid hormone secretion and promoted apoptosis in ovine ovarian GCs under high PRL concentrations by targeting *SREBF1*. These findings extend our understanding of GC dysfunction in reproductive disorders. The proliferation, apoptosis, and differentiation of GCs are critical for follicular development [21], where disrupted GC apoptosis can impair cell–cell communications between GCs and oocytes [22], ultimately leading to ovarian endocrine abnormalities [23,24]. While *MAPK12* has been recently implicated in GC apoptosis regulation through metabolomic analysis [25], our study provides the first evidence of a *MAPK12*-miRNA regulatory axis in this process.

The pathway enrichment analysis revealed that novel 58 regulates multiple apoptosis-related signaling cascades, including TNF, AMPK, MAPK, and FoxO pathways, which indicates that novel 58 targets genes, involvement in GC apoptosis, and steroidogenesis. Previous studies have shown that AMPK activation modulates steroidogenesis in PCOS GCs [26], while the AMPK/FOXO3 axis regulates cellular apoptosis [27]. Additionally, TNF triggers apoptosis through the NF-κB signaling pathway [28,29]. Our results demonstrate that *MAPK12* cooperates with miRNAs to regulate these pathways in GC apoptosis, representing a novel regulatory network in ovarian function.

In the present experiment, we found that novel 58-5p significantly decreased steroid hormone (E_2_ and P_4_) secretion and suppressed the expression of steroidogenic enzymes *CYP11A1*, *3β-HSD*, and *CYP19A1* in hyperprolactinemic GCs. GCs serve as the primary source of E_2_ and P_4_, where *CYP11A1* converts cholesterol to pregnenolone [30], *3β-HSD* catalyzes pregnenolone to P_4_, and *CYP19A1* aromatizes testosterone to E_2_ [31]. Thus, these results indicate that novel 58-5p may related to oocyte development. Moreover, the results of our experiment revealed that novel 58-5p simultaneously enhanced pro-apoptotic proteins (Caspase3, Bax) and suppressed anti-apoptotic BCL-2, consistent with previous reports linking miRNAs to apoptosis [32] and steroidogenesis [33]. These findings demonstrate that novel 58-5 coordinates both apoptotic and steroidogenic pathways in GCs.

Through protein interaction analysis, we identified SREBF1 as a central regulator interacting with SREBF2, ACACA, and PPARG in GC function. ACACA is a key factor in fatty acid metabolism and the interference of ACACA suppresses cellular proliferation and triggers apoptosis in LNCaP prostate cancer cells [34]. Similarly, PPARG may regulate granulosa cell proliferation, apoptosis, and steroid hormone secretion [35]. It was reported that *SREBF1* is related to the regulation of ovarian function and cell apoptosis [36,37]. In our experiment, cell apoptosis could be promoted and steroid secretion could be inhibited by interference of *SREBF1* in GCs with high PRL concentrations. This suggests that *SREBF1* and its interacting proteins may be closely related to apoptosis and the secretion of steroid hormones. Moreover, a mass of miRNAs (miR-153, miR-33, and miR-212) play a role by targeting *SREBF1* [38,39,40]. In this study, *SREBF1* as the target gene with the most significant difference targeted by novel 58 was selected for the subsequent experiment. Compared with the SREBF1-WT+58-5p group, the luciferase activity of the SREBF1-WT+58-5p group was significantly decreased, predicting that novel 58-5p may regulate apoptosis and steroid hormone secretion in ovine ovarian GCs by targeting *SREBF1*, and confirmed novel 58-5p has a negative regulatory effect on *SREBF1* mRNA expression. In addition, the ggccggctgggggattgccg sequence may be the target site of SREBF1 targeted by novel 58-5p. Importantly, our results establish *SREBF1* as a key mediator of novel 58-5p’s effects on GCs function under a high PRL concentration.

## 4. Materials and Methods

### 4.1. Experimental Design and Ovine Ovarian GCs Culture

The PRL concentration in sheep can reach more than 200 ng/mL during long exposure to sunshine. Our previous study found that high PRL concentrations (500 ng/mL PRL) promoted apoptosis and inhibited steroid hormone secretion in ovine ovarian GCs by upregulating *MAPK12* [13]. An effort to investigate the substantial molecular mechanisms and networks associated with MAPK12-induced apoptosis and the secretion of steroid hormones in GCs with high PRL concentrations was made. Ovine ovarian GCs preserved in liquid nitrogen were thawed and cultured in a medium consistent with our previous experiment descriptions [13]. Additionally, 500 ng/mL of PRL (Ovine PRL: PROSPEC, cyt-240) with 99% or greater purity was added to the medium as experiment treatment. Then, GCs in P group (*n* = 3, 500 ng/mL PRL) and P-10 group (*n* = 3, GCs treated with 500 ng/mL PRL were infected by lentiviruses carrying overexpressed sequences of *MAPK12*) were collected for whole-transcriptome analysis.

### 4.2. GCs Infection and RT-qPCR

#### 4.2.1. The GCs Model of Infection

The *MAPK12* overexpression lentiviruses were constructed using lentivirus-carrying expression vectors without inserts (pGWLV10-new vector, Figure S1) and were acquired from Jiangsu Genewiz Biotechnology Co., Ltd. (Suzhou, China). The lentiviruses containing overexpressed *MAPK12* sequences were used to infect GCs, and the lentiviruses with the pGWLV10-new vector were used to infect GCs as the negative control group (P-10-NC). To achieve overexpression, cells were plated at a density of 1 × 10^5^ cells per well in a 6-well plate. Subsequently, when the cells reached 70% confluence, they were infected with the lentivirus of each group at the optimal multiplicity of infection (MOI = 300). The success of overexpression was assessed by examining the expression of *MAPK12* after 48 h of incubation using RT-qPCR.

#### 4.2.2. RNA Extraction and RT-qPCR

Primers were designed according to conserved regions using Primer 5.0 software. The production of these primers was commissioned by Shanghai Sheng Gong Biotechnology Co., Ltd. (Shanghai, China). Detailed information is presented in Table 1. RNA extraction, reverse transcription, and RT-qPCR were performed according to a previous study [13].

### 4.3. RNA-Seq and Bioinformatics Analyses

#### 4.3.1. RNA Extraction, Library Construction and Quality Control

Ovine ovarian GCs subjected to the P (n = 3) and P-10 groups (n = 3) were used for RNA-Seq and the steps of RNA extraction were according to the methods described before [13]. The whole-transcriptome sequencing was conducted by Novogene Bioinformatics Technology (Beijing, China).

Adaptors for 3′ and 5′ were linked to the ends of small RNAs at 3′ and 5′, respectively. Subsequently, first-strand cDNA was generated following hybridization with the reverse transcription primer. The cDNA library with double-stranded DNA was prepared through PCR amplification. After purification and size selection, libraries containing inserts ranging from 18 to 40 base pairs were ready for Illumina SE50 sequencing. The library underwent assessment using Qubit and real-time PCR for quantification, alongside a bioanalyzer analysis for size distribution detection. Pooled libraries with quantified concentrations were be subjected to sequencing on Illumina platforms, based on the effective library concentration and the required data amount.

The raw data (raw reads) in the fastq format were initially subjected to processing through custom Perl and Python scripts (Python 2.7). In this stage, clean data (clean reads) were acquired by eliminating reads that contained poly-N, had 5′ adapter contaminants, lacked a 3′ adapter or the insert tag, contained poly A or T or G or C, and exhibited low-quality reads from the raw data. Simultaneously, calculations for Q20, Q30, and GC-content were performed on the raw data. Subsequently, a specific length range was selected from the clean reads for conducting all successive analyses.

#### 4.3.2. Alignment and Identification of Small RNA

Mapped small RNA tags were used to search for known miRNA [41]. The hairpin structure features of the miRNA precursor can be employed for predicting novel miRNAs, with mirdeep2 software (mirdeep 2_0_0_5). The identification of novel miRNAs was conducted utilizing miREvo (miREvo v1.1) [42] and mirdeep2 [43] software which involved detecting secondary structures, Dicer cleavage sites, and minimum free energies of unannotated small RNA tags identified in earlier steps.

#### 4.3.3. Differential Expression of miRNA and RT-qPCR Confirmation

Differential expression (DE) analysis between the P group and P-10 group was performed using the DESeq package in R software (1.8.3). miRNAs with a false discovery rate (FDR) < 0.05 and a |log2 (Fold Change)| > 1 (between two time points) were identified as significant DE miRNAs. Novel 49, novel 58, novel 100, and novel 105, related to cell apoptosis, underwent random selection for the validation of expression profiles extracted via RNA-Seq using the same RNA samples. Primers of novel 49, novel 58, novel 100, novel 105, and U6 were designed using the Stem loop method from the mature sequence and synthesized by Jiangsu ProbeGene Biotechnology Co., Ltd (Xuzhou, China). The miRNA primers used for RT-qPCR are listed in Table 2. Reverse transcription was performed using a Hifair III 1st Strand cDNA Synthesis Kit (11139ES10, Yeasen, Shanghai, China) according to the manufacturer’s instructions. The RNA reaction solution was incubated at 42 °C for 2 min and the configuration method is shown in Table S3. The 20 µL reaction mixture, consisted of 10× Hifair^®^ III Super Buffer (2 µL), Hifair^®^ III RT Enzyme Mix (1 µL), RT Primer (1 µL), RNA Reaction Liquid (15 µL), and RNase-free water (1 µL). The reaction lasted for 20 min with 5 min at 25 °C and 15 min at 55 °C. RT-qPCR was conducted by employing Hieff^®^ miRNA Universal qPCR SYBR Master Mix (Stem loop method) and the subsequent cycling protocol included an initial step of 5 min at 95 °C, succeeded by 40 cycles of denaturation for 10 s at 95 °C and annealing/extension for 30 s at 60 °C. The total reaction volume was 20 µL, comprising 10 µL of Hieff^®^ miRNA Universal qPCR SYBR Master Mix, 0.5 µL forward and reverse primers, 2 µL cDNA, and 7 µL ddH_2_O.

#### 4.3.4. Target Gene Prediction and Functional Enrichment Analysis

Predicting the target gene of ovine ovarian miRNAs was performed using miRanda (miRanda-3.3a) [44]. An enrichment analysis of Gene Ontology (GO) was applied to the candidate target genes of differentially expressed miRNAs, considering biological processes (BP), molecular function (MF), and cellular components (CC). The Goseq-based Wallenius [45] non-central hyper-geometric distribution was used for the enrichment analysis. The Kyoto Encyclopedia of Genomes (KEGG) pathway enrichment analysis of DE miRNA candidate target genes was performed using KOBAS (http://bioinfo.org/kobas (accessed on 5 May 2023)) [46] to examine the statistical enrichment of candidate target genes in the KEGG pathway. The statistical enrichment of the target gene candidates in the KEGG pathway was assessed using dedicated software (clusterProfiler 3.8.1).

#### 4.3.5. Construction of miRNAs-mRNAs-KEGG Pathway Interaction Network

The miRNAs-mRNAs-KEGG pathway interaction network was made using the Cytoscape (3.0) software. In this study, we found that novel 58 can regulate several candidate genes that participate in multiple apoptosis-related pathways. Among these candidate genes, *SREBF1* (sterol regulatory element binding factor 1) (*p* = 0.006) has the most significant difference with novel 58. Therefore, novel 58 and *SREBF1* were selected for the subsequent experiment.

### 4.4. Overexpression of Novel 58

#### 4.4.1. The Transfection of Novel 58 Related RNA

The negative control mimic (NC), novel 58-3p mimics, and novel 58-5p mimics were synthesized by Sangon Biotechnology Co., Ltd. (Shanghai, China). The experiment was grouped as follows, Con group: GCs with 500 ng/mL PRL; NC group: GCs in Con group transfected with the negative control of mimics; novel 58-3p group: GCs in Con group transfected with novel 58-3p mimics; and novel 58-5p group: GCs in Con group transfected with novel 58-5p mimics. Transfections were conducted using Lipofectamine 2000 (Invitrogen, Carlsbad, CA, USA) according to the manufacturer’s instructions. Then, the protein expression of SREBF1, BCL-2, Caspase 3, and Bax was analyzed in each group. Sequences for mimics NC, novel 58-3p mimics, and novel 58-5p mimics are shown in Table 3.

#### 4.4.2. Western Blotting

Total proteins were lysed with phenylmethanesulfonylfluoride (PMSF) and then subjected to 10% sodium dodecyl sulfate-polyacrylamide gel electrophoresis (SDS-PAGE) for separation. After the transfer onto polyvinylidene difluoride (PVDF) membranes (Bio-Rad, Hercules, CA, USA), the membranes underwent a standard blocking process with 5% non-fat milk for 2 h at room temperature. Following this, incubation with the secondary antibody (goat anti-rabbit, IgG, 1:500) took place for 1 h at the same temperature. The primary antibodies are as follows: SREBF1 (1:2000, 14088-1-AP, Proteintech, Wuhan, CN); BCL-2 (1:8000, 550599-1-AP, Proteintech, Wuhan, China); Bax (1:1500, 26593-1-AP, Proteintech, Wuhan, China); Caspase 3 (1:800, 19677-1-AP, Proteintech, Wuhan, China); and β-actin (20536-1-AP, Proteintech, Wuhan, China).

#### 4.4.3. Steroid Hormone Secretion and Related Gene Expression

The cell supernatant of each group was collected for the detection of estrogen and progesterone (E_2_ and P_4_). Hormone kits were obtained from Shanghai Enzyme Linked Biotechnology Co., Ltd. (Shanghai, China). Concentrations of P_4_ (NO.JLC10263; sensitivity > 0.1 ng/mL) and E_2_ (NO.JLC10385; sensitivity > 1 pg/mL) were measured according to the manufacturer’s description. The intraassay CV was 10%.

The expression of genes (*CYP11A1*, *CYP19A1*, and *3β-HSD*) related to steroid hormone secretion and synthesis was detected by RT-qPCR. Primers of *CYP11A1*, *CYP19A1*, and *3β-HSD* are listed in Table 1.

### 4.5. Dual-Luciferase Reporter Gene Assay

To further validate the binding association among novel 58-3p, novel 58-5p, and SREBF1, wild type SREBF1 (SREBF1-WT) and mutant SREBF1 (SREBF1-MUT) dual-luciferase reporter gene plasmids were constructed. SREBF1-WT and SREBF1-MUT were co-transfected into GCs with novel 58-3p mimics and novel 58-5p mimics, respectively, and grouped as follows: pmirGLO+NC (empty plasmid), pmirGLO+58-3p (pmirGLO was established with novel 58-3p), pmirGLO+58-5p (pmirGLO was established with novel 58-5p), SREBF1-WT+NC (wild type SREBF1), SREBF1-WT+58-3P (wild type SREBF1 was established with novel 58-3p), SREBF1-WT+58-5P (wild type SREBF1 was established with novel 58-5p), SREBF1-MUT-5P+NC (mutate SREBF1 at the 5p terminal binding site), SREBF1-MUT-5p+58-5p (mutant SREBF1 was established with novel 58-5p), SREBF1-MUT-3p+NC (mutate SREBF1 at the 3p terminal binding site) and SREBF1-MUT-3p+58-3p (mutant SREBF1 was established with novel 58-3p). The sequences of SREBF1-WT, SREBF1-MUT-5p, SREBF1-MUT-3p are shown in Supplementary S1. Luciferase reporter gene vector pmirGLO and all synthetic plasmids were obtained from Probegene Biotechnology Co., Ltd. (Xuzhou, China).

GCs were seeded in 96-well culture plates at a density of 5 × 10^3^ per well and treated for 24 h. Then, 200 ng of pmirGLO recombinant vector and 100 nM mimics or the negative control (NC) were co-transfected into GCs using Lipofectamine 2000 (Invitrogen, Carlsbad, CA, USA) according to the manufacturer’s instructions. The GCs were cultured at 37 °C with 5% CO_2_ and three repetitions per group. The function of the dual-luciferase enzymes was assessed utilizing the dual-luciferase reporter assay system (Promega, Madison, WI, USA) 48 h post-transfection. The outcomes are presented as the means of the firefly luciferase/Renilla luciferase activity ratio.

### 4.6. Functional Validation of SREBF1

#### 4.6.1. Bioanalysis of SREBF1

Bioinformatic analyses proceeded for the CDs region of Ovis *SREBF1*. The species names and accession numbers are shown in Table S4, and the main bioinformatic tools used are presented in Table S5.

#### 4.6.2. Gene Editing of SREBF1 in GCs with a High PRL Concentration

The interference and overexpressed sequences of *SREBF1* were designed and synthesized by Jiangsu ProbeGene Biotechnology Co., Ltd. The sequences of siRNAs are shown in Table 4 and the overexpression sequences are shown in Supplementary S2. The overexpression expression vector (pcDNA3.1-SREBF1) was constructed using the overexpressed sequence of *SREBF1*. GCs with high PRL concentrations were transfected by siRNAs and overexpressed synthetic plasmids using Lipofectamine 2000 (Invitrogen, Carlsbad, CA, USA) according to the manufacturer’s protocol (Con group: GCs with 500 ng/mL PRL, KD group: GCs in Con group transfected with interference sequence of *SREBF1*, OE group: GCs in the Con group transfected with overexpressed sequence of *SREBF1*). Samples were collected 48 h after transfection for RT-qPCR and WB to verify whether the interference and overexpression were successful. The primer of *SREBF1* is listed in Table 1.

#### 4.6.3. Apoptosis Assay

GCs in the Con, KD, and OE groups were inoculated into 6-well plates at a density of 1 × 10^5^ per well and incubated for 24 h. The cells were washed with PBS and resuspended in a 1 Annexin V binding buffer. The resultant cultures were incubated in the dark for 15 min with Annexin V-FITC and PI reagents. Ultimately, apoptosis was determined in 1 h using a BD FACSCanto II Flow Cytometer (Becton Dickinson, New Jersey, USA). In addition, each experiment was performed in triplicate.

The gene expression (*Bax*, *Bcl-2*, and *Caspase3*) related to apoptosis was detected by RT-qPCR. Primers of *Bax*, *Bcl-2*, and *Caspase3* are listed in Table 1.

#### 4.6.4. Steroid Hormones Secretion

The cell supernatant of the Con, KD, and OE groups was collected for the detection of E_2_ and P_4_ according to the method in Section 4.4.3.

### 4.7. Statistical Analysis

The results of the experiment were statistically analyzed using SPSS software version 22.0. The data were expressed as the means obtained through least squares analysis, along with corresponding standard errors. The outcomes acquired from the RT-PCR were subjected to statistical analysis using a *t*-test to compare the P group with the P-10 group. The analysis of steroid hormone expression, genes, and proteins, as well as the dual-luciferase reporter gene assay among different groups, was conducted using a one-way ANOVA. Statistical differences were evaluated through a Duncan’s test. A significant difference is denoted by *p* < 0.05. Data visualization and plotting were performed using GraphPad Prism 9.0 software.

## 5. Conclusions

Novel 58-5p suppressed steroid hormone secretion and induced apoptosis in PRL-treated ovine granulosa cells by directly targeting SREBF1. SREBF1 exhibited opposite effects and formed a negative feedback loop with novel 58-5p through the binding site ggccggctgggggattgccg.

## Figures and Tables

**Table 1:** The primer information for the RT-qPCR assay.

Gene Name	Primer Sequence (5′-3′)	Tm (°C)
*P53*	F: AAGCAGGGCTCATTCTAGCC R: GGCCCTTCTCTCTTGAGCAT	60
*MAPK12*	F: GCAGGCAGACAGCGAGAT R: GGTCAGGACGGAGGCAAA	62
*CYP11A1*	F: GTTTCGCTTTGCCTTTGAGTC R: ACAGTTCTGGAGGGAGGTTGA	60
*3β-HSD*	F: CAGTCTATGTTGGCAATGTGGC R: CGGTTGAAGCAGGGGTGGTAT	60
*CYP19A1*	F: GCTTTTGGAAGTGCTGAACCC R: CATGCCGATGAACTGCAACC	60
*SREBF1*	F: TTGGAGCGAGCACTGAATTG R: GTTCAGCAGCTGCAGGTATC	60
*BCL-2*	F: CCTTTGTGGAGCTGTATGGC R: CCTTTGTGGAGCTGTATGGC	60
*Bax*	F: CATCATGGGCTGGACATTGG R: GTGGGTGTCCCAAAGTAGGA	60
*Caspase3*	F: TTCAGAGGGGACTGTTGCAG R: CAGTCCAGTTCTGTGCCTCG	60
*β-actin*	F: TCAGCAAGCAGGAGTACGAC R: GGGTGTAACGCAGCTAACAG	60

**Table 2:** Sequence of primers.

Name	Sequence
novel_58-5p	novel_58-5p RT: GTCGTATCCAGTGCAGGGTCCGAGGTATTCGCACTGGATACGACAACAAC
	novel_58-5p (F): GCGTGGCAGTGTCTTAGCTG
novel_58-3p	novel_58-3p RT: GTCGTATCCAGTGCAGGGTCCGAGGTATTCGCACTGGATACGACTAGGGC
	novel_58-3p (F): CGCGCAATCAGCAAGTATACT
novel_49-5p	novel_49-5p RT: GTCGTATCCAGTGCAGGGTCCGAGGTATTCGCACTGGATACGACGGAGGC
	novel_49-5p (F): GCGTATTGCACTCGTCCCG
novel_49-3p	novel_49-3p RT: GTCGTATCCAGTGCAGGGTCCGAGGTATTCGCACTGGATACGACACACTG
	novel_49-3p (F): GGGACGGGACGCGGTG
novel_100-5p	novel_100-5p RT: GTCGTATCCAGTGCAGGGTCCGAGGTATTCGCACTGGATACGACAGTCAG
	novel_100-5p (F): GCGCGTGGATAACGCGT
novel_100-3p	novel_100-3p RT: GTCGTATCCAGTGCAGGGTCCGAGGTATTCGCACTGGATACGACAAAACA
	novel_100-3p (F): CGCGGGGAGACGCGTG
novel_105-5p	novel_105-5p RT: GTCGTATCCAGTGCAGGGTCCGAGGTATTCGCACTGGATACGACGCAATC
	novel_105-5p (F): CGCGAGGCAGTGTAGTTAGCT
novel_105-3p	novel_105-3p RT: GTCGTATCCAGTGCAGGGTCCGAGGTATTCGCACTGGATACGACCCTGGC
	novel_105-3p (F): CGCGAATCACTAACCACACG
U6	U6 (F): GCGCGCTCGCTTCGGC
	U6 (R): AGTGCAGGGTCCGAGGTATT

Note: The green-highlighted sequence represents the 6-nucleotide complement to the miRNA 3′ end.

**Table 3:** The sequences of novel 58-related RNA.

Name	Sequence (5′-3′)
mimics NC	Sense: UUGUACUACACAAAAGUACUG Antisense: GUACUUUUGUGUAGUACAAUU
Novel 58-3p mimics	Sense: CAAUCAGCAAGUAUACUGCCCUA Antisense: GGGCAGUAUACUUGCUGAUUGUU
Novel 58-5p mimics	Sense: UGGCAGUGUCUUAGCUGGUUGUU Antisense: CAACCAGCUAAGACACUGCCAUU

Note: The red and blue sequences are designed as complementary pairs to form the predicted secondary structure.

**Table 4:** Primer sequences of siRNA.

Name	Sequence (5′-3′)
siRNA1	F: GCCGCGGAACCAUGGACUGCACGUU R: AACGUGCAGUCCAUGGUUCCGCGGC
siRNA2	F: UCCUUCCACGAUGAGCUCCUCACUU R: AAGUGAGGAGCUCAUCGUGGAAGGA
siRNA3	F: CGGAGCUCUCCUGCCGCAGAGUGUU R: AACACUCUGCGGCAGGAGAGCUCCG

## Data Availability

All datasets generated during and analyzed during the current study are available from the corresponding author (zhangyingjie66@126.com) upon reasonable request.

## Supplementary Materials

The following supporting information can be downloaded at: https://www.mdpi.com/article/10.3390/ijms26020576/s1.
