# Supplementary material for: Identification of Novel 58-5p and SREBF1 Interaction and Effects on Apoptosis of Ovine Ovarian Granulosa Cell
Source: Int J Mol Sci. 2025 Jan 11;26(2):576. doi: 10.3390/ijms26020576 (PMC11765093; doi:10.3390/ijms26020576)
Supplement: Supplementary file 1 [file ijms-26-00576-s001.zip › Supplementary 1.pdf]

Supplementary 1

1. SREBF1-WT（The blue marked sequence is the predicted novel 58-5p target, and the box sequence is the predicted novel 58-3p target）

actcctgccggcgggcctgccccagagcctggtctccgtatcagcggccgagagcagcgccggagatccggggctcacg
cccagcccacggactgcacggcccctgggggggctggagaccctcggagacgtctgctcttgacctgcgggcctccccagc
ccgctcccgcactcggcgcgcggtggccaggatagtgctggcccctggtggccggctgggggattgccgggggcctgccg
ccgctggatgtcacttgctcccagtgacacccgctcctgggtgtcatgggcccccagtccgcagctttcaccctccctccaga
gagaagagaggggtgcatttcaccgagccgagggaccctacccccggtgcctccctcccatctagggagacctgtgcata
gtgtagatcggagtgcaccagcctcctggcctccaaggctcaagcgttactttgccttttgcagactttattttcataggttgag
aagttttgtacagagaataaaaaatgaaattatttataa

2. SREBF1-MUT-5p

actcctgccggcgggcctgccccagagcctggtctccgtatcagcggccgagagcagcgccggagatccggggctcacg
cccagcccacggactgcacggcccctgggggggctggagaccctcggagacgtctgctcttgacctgcgggcctccccagc
ccgctcccgcactcggcgcgcggtggccaggatagtgctggcccctggtgggcctgccgccgctggatgtcacttgctccc
agtgacacccgctcctgggtgtcatgggcccccagtccgcagctttcaccctccctccagagagaagagaggggtgcattt
caccgagccgagggaccctacccccggtgcctccctcccatctagggagacctgtgcatagtgtagatcggagtgcacca
gcctcctggcctccaaggctcaagcgttactttgccttttgcagactttattttcataggttgagaagttttgtacagagaataa
aaaatgaaattatttataa

3. SREBF1-MUT-3p

actcctgccggcgggcctgccccagagcctggtctccgtatcagcggccgagagcagcgccggagatccggggctcacg
cccagcccacggactgcacggcccctgggggggctggagaccctcggagacgtctgctcttgacctgcgggcctccccagc
ccgctcccgcactcggcgcgcggtggccaggatagtgctggcccctggtggccggctgggggattgccccagtgacacc
cgctcctgggtgtcatgggcccccagtccgcagctttcaccctccctccagagagaagagaggggtgcatttcaccgagcc
gagggaccctacccccggtgcctccctcccatctagggagacctgtgcatagtgtagatcggagtgcaccagcctcctggc
ctccaaggctcaagcgttactttgccttttgcagactttattttcataggttgagaagttttgtacagagaataaaaaatgaaat
tatttataa
