# Supplementary material for: Identification of Novel 58-5p and SREBF1 Interaction and Effects on Apoptosis of Ovine Ovarian Granulosa Cell
Source: Int J Mol Sci. 2025 Jan 11;26(2):576. doi: 10.3390/ijms26020576 (PMC11765093; doi:10.3390/ijms26020576)
Supplement: Supplementary file 1 [file ijms-26-00576-s001.zip › Supplementary 2.pdf]

Supplementary 2

SREBF1-ATGGACGAGCCACCCTTCAACGAAGCGGCCTTGGAGCTGGCGCTGGCCGA
GCCGTGCGAGCTGGACGCGGCGCTGCTGACCGACATAGAAGGTGGGTCAGGGCCACT
GGGCTCCGCGCGCGGGCGGCGCCGGGCGCGGGGCGCGGAGGCGCCCCGAGTGGCGA
CGGGCCCGGGCCGCGGAACCATGGACTGCACGTTCGAAGACATGCTTCAGCTCATCAA
CAACCAAGACAGCGACTTCCCGGGCCTGTTTGACCCGCCCTACGCTGGGGGTGGAGC
AGGGACCACAGACCCTGCCAGTCCCGATGCCAGCTCCCCAGGCAGCCTGTCCCCACCT
CCTTCCACGATGAGCTCCTCACTTGAAGGCTTCCTGGGGGCGACCAAGGCGACACCCC
CACCCTTGTCTCCTCCCCAGCCTGCACCCACCCCCCTGAAGATGTACCCATCTGTGCCT
GCCTTCTCCCCGGGGCCTGGGATCAAGGAGGAGCCAGCGCCCCTTACTATCCTCCAGC
CCACCCCACCCCAGCCCCTGCCCGGAGCTCTCCTGCCGCAGAGTGTTGCGGCCACCAC
CCCACCGCAGTTCAGCTCTGCCCCCATTGTAGGCTACCCCAGCCCTCCGGGAGGCTTC
TCCACAGGGACCCCTCCGGGGAGCAGCTCGCAGCCACTGGCTGGCCCACCACTGGCT
TCCCTGCCAGGGGTCCCGCCTGTCTCCTTGCACAGCCAGGTTCAGAGTGCGGCCCCCC
AGCAGCTGTTGACAGCCATAGCCACCCCCACGGTGGCCCCTGGAGCAACTGCTGTGAC
CTCCCAGATACAGCAGGTCCCGGTCCTGCTGCAGCCCCACTTCATCAAGGCAGACTCG
TTGCTCCTGACGACCATGAAAACAGATGTGGGAGCCCCCTTGAAAGCGGCGGGCATCC
GCTCCCTGGGCCCTGGCACTGCTATGCAGGCAGCACCCTTGCAGACCCTGGTGAGTGG
CGGGGCCATCCTGGCCACTGTGCCACTGGTAGTGGACACTGACAAGCTGCCCATCAAC
CGACTTGCCGGTGGCAAGGCTCCAGGCTCGGCGCAGAGCCGCGGCGAGAAGCGTACA
GCCCACAACGCCATCGAGAAACGCTACCGCTCTTCCATCAATGACAAGATCGTTGAGC
TCAAGGACCTGGTGGTGGGCACCGAGGCCAAGTTGAATAAATCTGCCGTCTTGCGCAA
AGCCATCGACTACATCCGCTTCCTTCAGCACAGCAACCAGAAGCTCAAGCAGGAGAA
CCTGAGTCTGCGCACTGCTGTCCATAAAAGCAAATCACTGAAGGACCTGGTGTCGGCC
TGCGGCAGTGGAGGTAGCACAGATGTGCCCATGGAGGGCATGAAGCCCGAGGTGGTG
GACACCCTGAGCCCCCCCCCTCAGACGCCGGCTCACCCTCCCAGAGCAGCCCCTTGT
CCCTTGGCAGCAGGGGCAGTAGCAGCGGTGGAAGTGGCAGTGACTCGGAGCCTGACA
GCCCGGTCTTTGAGGACGGCCAGGTGAATCCAGAGCTGCTGCCCGCCCCCACAGCC
AGGGCATGCTGGACCGCTCTCGCCTGGCCCTGTGTGCGCTCGTCTTCCTCTGTCTCTCC
TGCAACCCCTTGGCCTCCCTGCTGGGTAGCCGGGGTCCTGCTGGCCCCTCCGACACCA
CCAGCATCAACCACCGTCCTGGGCGCAGCATGCTGGGTGCTGAGGGCAGAGATGGCC
CTGGCTGGGCCCCGTGGCTGCTGCCCCCACTGGTCTGGCTGATGAATGGGCTGCTGGT
GCTCTTCTCCTTGGCGCTTCTCTTTGTCTATGGAGAACCAGTCACTCGGCCCCACTCGC
GCCCTGCCGTGCACTTCTGGAGGCATCGCAAGCAGGCCGACCTGGACCTGGCCAGGG

GGGACTTTGCCCAGGCTGCCCAGCAGCTGTGGCTGGCCCTGCGGGCCTTGGGCCGGC
CTCTGCCCACCTCCCACCTGGACCTGGCCTGCAGCCTGCTTTGGAGCCTCATCCGCCA
CCTGCTGCAGCGTCTCTGGGTGGGCCGCTGGCTGGCCGGCTGGGCAGGGGGCCTACG
GAGGGACAGGGCCCTACAGGCAGACGCTCGCACCAGTGCCCGCGATGCGGCCCTCGT
CTACCACAAGCTGCACCAGCTGCACACCATGGGGAAGTACTCAGGTGGGCACCTCGCT
GCTGCCAACCTGGCGCTGAGTGCCCTGAACCTGGCCGAGTGTGCGGGAGATGCTGTG
TCCGTGGCCACACTGGCTGAGATCTACGTGGCCGCCGCGCTCAGGGTCAAGGCCAGTC
TGCCCCGGGCCTTGCATTTTCTGACACGCTTCTTCCTGAGCAGTGCCCGCCAGGCCTG
CCTGGCACAGAGTGGCTCAGTGCCCCTTGCCATGCAGTGGCTCTGCCACCCTGTGGGC
CACCGTTTCTTCGTGGATGGCAACTGGGCCCTGTGCAGCGCCCCGAGGGACAGCTTGT
ACAGCGTGGCTGGGAACCCAGTGGATCCCCTGGCCCAGGTGACTCAACTGTTCCGCG
AACATCTGTTGGAGCGAGCACTGAATTGCGTGGCCCAGCCCAGCCCTAGCCCTGGATC
AGCCGAGGGGGACAAGGAGTTCTCAGATGCCCTCGGATACCTGCAGCTGCTGAACAG
CTGTTCGGATATGGCCGGAGCTCCTGCCTGCAGCTTCTCCATCAGCTCCAGCATGGCTG
CCACCCCCGGCACAGACCCGGTGGCCAAGTGGTGGGCCTCTCTGACAGCTGTGGTGA
CCCACTGGCTTCGGCGGGATGAGGAGGCAGCTGAGAGGCTGTACCCACTGGTGGAGC
ACCTGCCCCGTGCCCTGCAGGAGTCCGAGAAACCCCTGCCCAGGGCGGCTCTGCACT
CCTTCAAGGCTGCCCGGGCCATCCTAGGCCGCGGGAAGGCTGAGTCTGGCCCAGCCA
GCCTGGTGATGTGTGAGAAGGCCAGTGGGTACCTGCAGGACAGCCTGGCCACCACAC
CAGCTGACAGCTCCATTGACAAGGCCATGCAGCTGCTCCTGTGTGACCTGCTCCTTGT
GGCGCGCACCAGCCTCTGGCAGCAGCAGAAGCTGCCGGCACCCACCCAGGCCTCGCA
GGGCCCTGGAGGTGGGGCCCAGGCCTCTGCCCTCGAGCTTCGTGGTTTCCAGAGGGA
CTTGAGTGGCCTGAGGCGTCTGGCACAGAATGTCCGGCCTGCCATGCGGAGGGTATTC
CTACATGAAGCCACTGCCCGACTGATGGCAGGGGCCAGCCCAGCGCGGACACACCAG
CTTCTGGACCGTAGCCTGAGGAGGAGGGTCGGCCCCTGCAAAGGAGGCGCGGCGGTG
GAGCTGGAGTCGCGGCCCACGAGGCGGGAGCAGGCCGAGGCTTTGCTGCTGGCCTCC
TGCTACCTGCCGCCTGGCTTCCTGTCGGCGCCCGGGCAGCGCGTGGGCATGCTGGCCG
AGGCGGCGCGCACGCTCGAGAAGATTGGTGACCGCCGGCTGCTGCACGACTGTCAGC
AGATGCTCATGCGCCTGGGCGGCGGGACCACTGTGACCTCCAGCTAG
