# Supplementary material for: Identification of Novel 58-5p and SREBF1 Interaction and Effects on Apoptosis of Ovine Ovarian Granulosa Cell
Source: Int J Mol Sci. 2025 Jan 11;26(2):576. doi: 10.3390/ijms26020576 (PMC11765093; doi:10.3390/ijms26020576)
Supplement: Supplementary file 1 [file ijms-26-00576-s001.zip › Table S1 Quality control of the samples.pdf]

Table S1 Quality control of the samples

Sample	Total Reads	N% > 10%	5' adapter contamine	3' adapter null or insert null	With ployA/T/G/C	Clean Reads
P1	11379509	0 (0.00%)	792 (0.01%)	122799 (1.08%)	3177 (0.03%)	11252741(98.89%)
P2	11353165	1095 (0.01%)	907 (0.01%)	96746 (0.85%)	2923 (0.03%)	11251494(99.10%)
P3	11753943	1160 (0.01%)	2040 (0.02%)	140668 (1.20%)	4473 (0.04%)	11605602(98.74%)
P_10-1	11111878	0 (0.00%)	1031 (0.01%)	151962 (1.37%)	2727 (0.02%)	10956158(98.60%)
P_10-2	11634454	0 (0.00%)	676 (0.01%)	125832 (1.08%)	2217 (0.02%)	11505729(98.89%)
P_10-3	11561620	0 (0.00%)	769 (0.01%)	75634 (0.65%)	2751 (0.02%)	11482466(99.32%)
