# Supplementary material for: Identification of Novel 58-5p and SREBF1 Interaction and Effects on Apoptosis of Ovine Ovarian Granulosa Cell
Source: Int J Mol Sci. 2025 Jan 11;26(2):576. doi: 10.3390/ijms26020576 (PMC11765093; doi:10.3390/ijms26020576)
Supplement: Supplementary file 1 [file ijms-26-00576-s001.zip › Table S2 Differentially expressed miRNAs.pdf]

Table S2 Differentially expressed miRNAs

siRNA	P-10 readcount	P readcount	P-value
novel_58	279.7280854	157.4218311	2.45E-05
oar-miR-16b	1036.254221	1425.049025	0.002669576
novel_387	62.67016718	116.9529908	0.002896342
oar-let-7a	36702.82471	47919.9118	0.004353569
novel_59	65.09721378	108.4552727	0.007042738
oar-miR-143	13978.75569	19217.18788	0.009621626
oar-miR-221	3409.305334	4225.884408	0.009790075
novel_55	241.8035539	352.2044339	0.013196322
novel_105	37.28930078	16.87571617	0.014634573
novel_89	36.6796168	65.85252303	0.023725417
oar-let-7d	1556.753152	2052.73564	0.026470367
novel_83	93.44855271	59.68938344	0.027928942
novel_100	27.6665547	52.57369532	0.04481298
novel_49	402.2478138	312.6218589	0.046975067
