# Supplementary material for: Identification of Novel 58-5p and SREBF1 Interaction and Effects on Apoptosis of Ovine Ovarian Granulosa Cell
Source: Int J Mol Sci. 2025 Jan 11;26(2):576. doi: 10.3390/ijms26020576 (PMC11765093; doi:10.3390/ijms26020576)
Supplement: Supplementary file 1 [file ijms-26-00576-s001.zip › Table S3 RNA Reaction Solution.pdf]

Table S3 RNA Reaction Solution

Component	Dose
RNase-free H_2O	To 15 μL
5×gDNA Digester Mix	3 μL
Total RNA	10 pg-5 μg
