# Supplementary material for: Identification of Novel 58-5p and SREBF1 Interaction and Effects on Apoptosis of Ovine Ovarian Granulosa Cell
Source: Int J Mol Sci. 2025 Jan 11;26(2):576. doi: 10.3390/ijms26020576 (PMC11765093; doi:10.3390/ijms26020576)
Supplement: Supplementary file 1 [file ijms-26-00576-s001.zip › Table S4 Species names and accessing numbers.pdf]

Table S4 Species names and accessing numbers

Species	*Ovis*	*Capra hircus*	*Bos taurus*	*Sus scrofa*	*Mus musculus*	*Homo sapiens*	*Gallus gallus*
Accessing Number	XM_ 027974786.2	NM_ 001285755.1	NM_ 001285755.1	NM_ 214157.1	NM_ 001313979.1	NM_ 001005291.3	NM_ 204126.3
