# Supplementary material for: Identification of Novel 58-5p and SREBF1 Interaction and Effects on Apoptosis of Ovine Ovarian Granulosa Cell
Source: Int J Mol Sci. 2025 Jan 11;26(2):576. doi: 10.3390/ijms26020576 (PMC11765093; doi:10.3390/ijms26020576)
Supplement: Supplementary file 1 [file ijms-26-00576-s001.zip › Table S5 Bioinformatics related software and its f.pdf]

Table S5 Bioinformatics related software and its functions

Software	URL	Purpose
ProtScale	https://web.expasy.org/protscale/	Prediction of hydrophobicity of protein
SignalP 4.1	http://www.cbs.dtu.dk/services/SignalP-4.1/	Prediction of protein signal peptide
TMHMM	http://www.cbs.dtu.dk/services/TMHMM/	Prediction of transmembrane region
NetPhos 3.1	https://services.healthtech.dtu.dk/service.php?NetPhos-3.1	Prediction of phosphorylation site score of protein
SOPMA	https://npsa-prabi.ibcp.fr/cgi-bin/npsa_automat.pl?pagea_ sopma.html	Prediction of protein secondary structure
SWISS-MODEL	https://swissmodel.expasy.org/comparison/	Prediction of protein tertiary structure
STRING 11.5	https://cn.string-db.org/	Analysis of the relationship between protein interaction network
$GSDS_{2.0}$	http://gsds.gao-lab.org/	Analysis of protein domain
