# Supplementary figures and images for: Identification of Novel 58-5p and SREBF1 Interaction and Effects on Apoptosis of Ovine Ovarian Granulosa Cell
Source: Int J Mol Sci. 2025 Jan 11;26(2):576. doi: 10.3390/ijms26020576 (PMC11765093; doi:10.3390/ijms26020576)

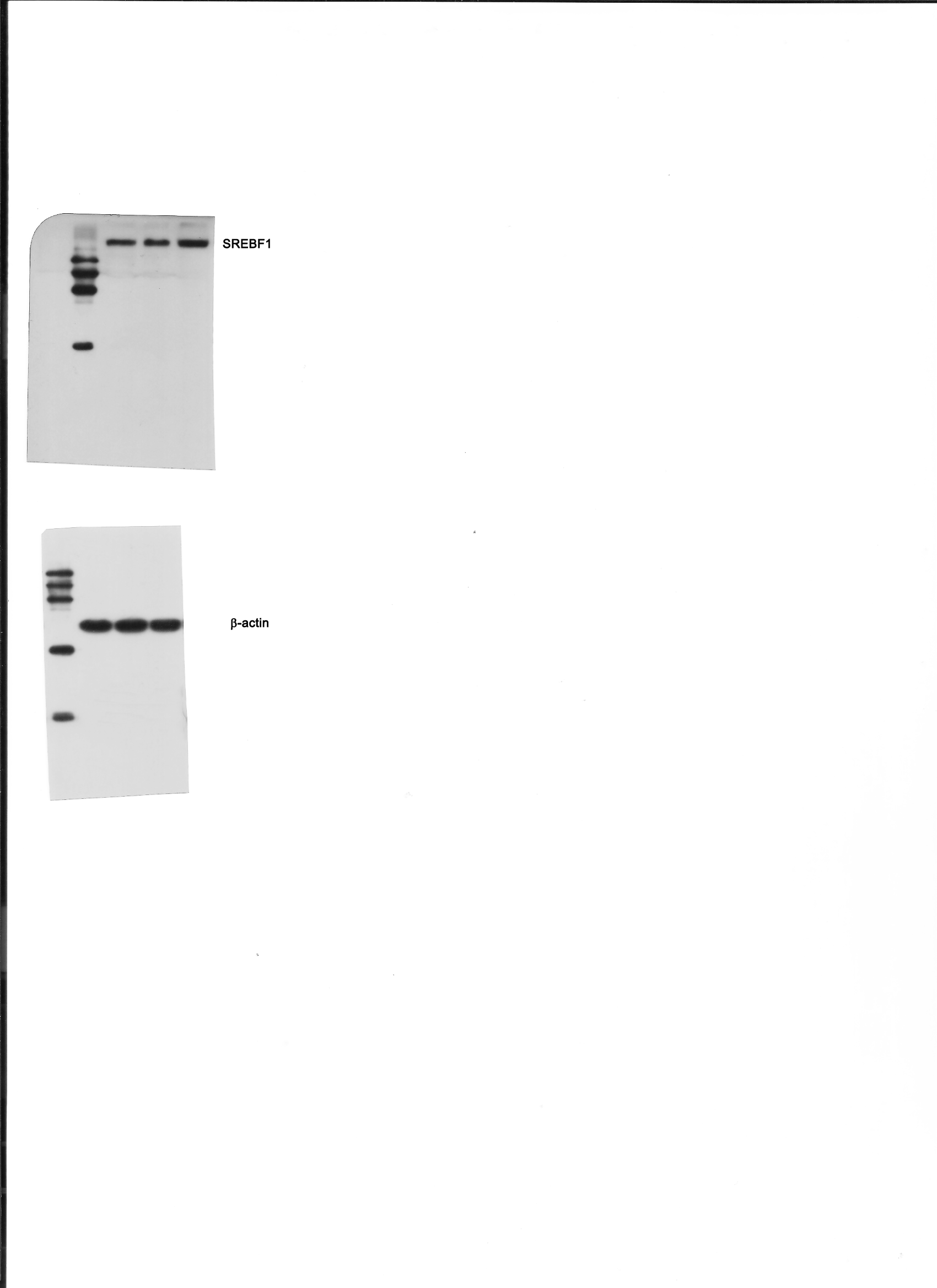

Supplement: Supplementary file 1 [file ijms-26-00576-s001.zip › WB/SREBF1.tif]

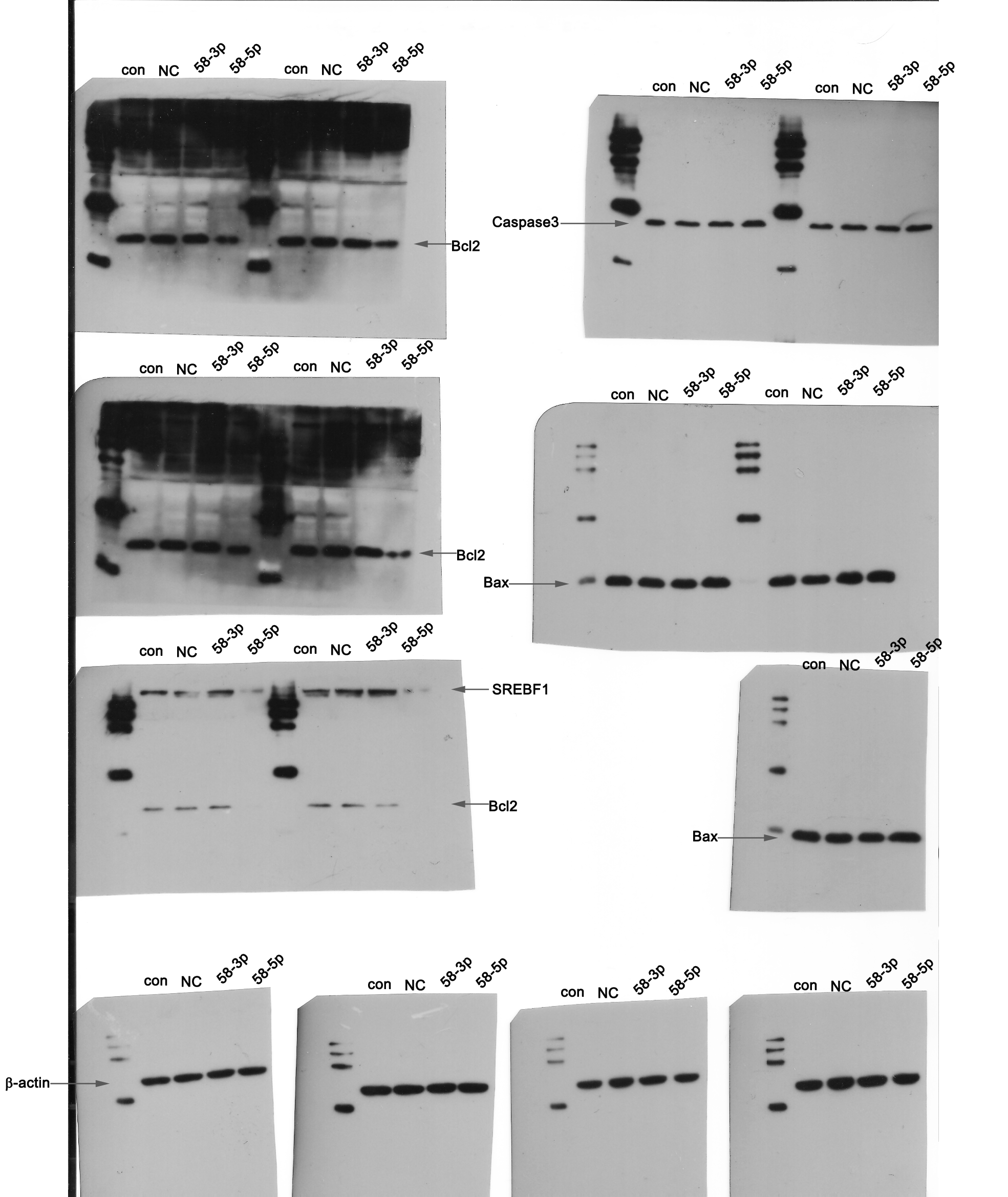

Supplement: Supplementary file 1 [file ijms-26-00576-s001.zip › WB/WB.tif]
